# 4P: fast computing of population genetics statistics from large DNA polymorphism panels

**DOI:** 10.1002/ece3.1261

**Published:** 2014-12-11

**Authors:** Andrea Benazzo, Alex Panziera, Giorgio Bertorelle

**Affiliations:** 1 Department of Life Sciences and Biotechnology, University of Ferraravia L. Borsari, 46, 44100, Ferrara, Italy; 2 Department of Biodiversity and Molecular Ecology, Fondazione Edmund Machvia E. Mach 1, 38010 S, Michele all'Adige, Italy

**Keywords:** Allelic spectrum, *F*_st_, genetic indicators, genetic variation, NGS, software

## Abstract

Massive DNA sequencing has significantly increased the amount of data available for population genetics and molecular ecology studies. However, the parallel computation of simple statistics within and between populations from large panels of polymorphic sites is not yet available, making the exploratory analyses of a set or subset of data a very laborious task. Here, we present *4P* (parallel processing of polymorphism panels), a stand-alone software program for the rapid computation of genetic variation statistics (including the joint frequency spectrum) from millions of DNA variants in multiple individuals and multiple populations. It handles a standard input file format commonly used to store DNA variation from empirical or simulation experiments. The computational performance of *4P* was evaluated using large SNP (single nucleotide polymorphism) datasets from human genomes or obtained by simulations. *4P* was faster or much faster than other comparable programs, and the impact of parallel computing using multicore computers or servers was evident. *4P* is a useful tool for biologists who need a simple and rapid computer program to run exploratory population genetics analyses in large panels of genomic data. It is also particularly suitable to analyze multiple data sets produced in simulation studies. Unix, Windows, and MacOs versions are provided, as well as the source code for easier pipeline implementations.

## Introduction

Next-generation sequencing (NGS) technologies, now in their third generation of systems, have led to a dramatic increase of polymorphism's data available in model and nonmodel species. For example, about 38 million single nucleotide polymorphisms (or SNPs) have been identified in the human genome, and data are now available at each of them for more than one thousand individuals (Abecasis et al. 2012). In nonmodel organisms, where a reference genome is not available, several thousands of SNPs can be now isolated and typed using specific protocols (Davey et al. 2011). All this genetic data can be used to obtain precise answers to old questions, for example, regarding the levels of population differentiation (Keller et al. 2013; Ogden et al. 2013) or the admixture rates (Hohenlohe et al. 2013) and are essential for the identification of genomic regions responsible of adaptation and speciation (The Heliconius Genome Consortium 2012; Hess et al. 2013; Wagner et al. 2013). This continuously accelerating trend is challenging the methods for data analysis, both computationally and theoretically. Basic statistics of genetic variation must now be computed for millions (rather than tens) of markers, their distributions can be estimated empirically from the data, and complex inferential methods require faster algorithms and computer clusters.

Statistics estimating the levels of genetic variation within and between populations are usually computed in exploratory analyses of real data, or for summarizing genetic patterns in large numbers of simulated data sets. When millions of loci are involved, however, the computer time required to complete a single analysis with the available software packages is unfeasibly long. Programs like *stacks* (Catchen et al. 2013) and *PLINK* (Purcell et al. 2007), designed for specific aims such as analyzing RAD tag sequencing data or performing association studies, respectively, use serial computations and are not optimized for speed. *Adegenet* (Jombart and Ahmed 2011) and *PopGenome* (Pfeifer et al. 2014) implement tools for the analysis of genome-wide SNPs, but they are R packages with only a few functions that can be executed in parallel. These difficulties mean that ad hoc scripts must be developed, and/or data sets have to be partitioned before the analysis.

Here, we introduce *4P* (parallel processing of polymorphisms panels), a new OpenMP-accelerated software written in ANSI C. *4P* was specifically designed to rapidly compute common population genetic statistics from SNP data sets. Our implementation ensures a notable increase in computational speed especially in shared memory systems such as multicore personal computers or servers.

## General features and supported input formats

A panel of SNPs can be simply described as an *N* × *M* matrix, where *N* corresponds to the number of individuals (or homologous chromosomes in diploids) and M to the number of SNPs. This is the most convenient way to store the data in dynamic memory, considering the ability of the C language to handle two-dimensional matrices and the possibility to directly apply parallelization techniques. In our implementation, as all the statistics in *4P* do not use information across loci, we used the OpenMP API to assign a certain number of columns (SNPs) to each core, thus allowing parallel computation by data partition. *4P* is not limited to any size of *N* or *M*. The main limiting factor is the amount of RAM available on the system, due to the fact that the matrix is loaded before starting the computation. *4P* can read SNP files created by other programs commonly used to identify or validate polymorphisms, such as *PLINK* and *BAM*/*SAM*/*VCF*-tools (Li et al. 2009; Barnett et al. 2011; Danecek et al. 2011). Because reading large data files from the disk is highly time-consuming, we implemented optimized routines for data import from files with the formats *ped*/*map* and *vcf* (v4.1). The output of *fastsimcoal* (Excoffier and Foll 2011; Excoffier et al. 2013), a commonly used coalescent simulator of genomic data, is also supported as a 4P input file. *4P* can be easily integrated in pipelines that require a preliminary computation of summary statistics from real or simulated data (e.g., when using *dadi*, Gutenkunst et al. 2009, or when performing an approximate Bayesian computation analysis). Additional details are provided in the online manual of *4P*.

## Implemented statistics

*4P* computes several statistics useful to summarize and explore genetic diversity within and between populations. Output is provided for each locus, or as means and variances across loci. The number of alleles for each locus can vary between 1 (monomorphic locus) and 4 (tetra-allelic locus).

The within-populations statistics implemented in *4P* are: allele frequencies, observed and expected heterozygosities, and single population and multipopulation allele frequency spectrum. The between-populations statistics implemented in *4P* are: *G*_st_ (three formulations, Nei 1973; Nei and Chesser 1983; and Hedrick 2005), Jost's *D* (Jost 2008), and the classical *F*_st_ (Weir and Cockerham 1984). In addition, *4P* computes the proportion of shared alleles between all pairs of individuals, from the same or from different populations.

## Computational performances

We analyzed the performance of *4P* using a dual 6-core Intel Xeon® X5650 running at 2.66 GHz with 32GB of RAM. The SNP data sets (stored in *arp* formatted files) were generated by simulation using the software *fastsimcoal* 1.0 (Excoffier and Foll 2011), assuming a model with two populations separated by 1000 generations and composed of 500 diploid individuals each.

**Figure 1:**
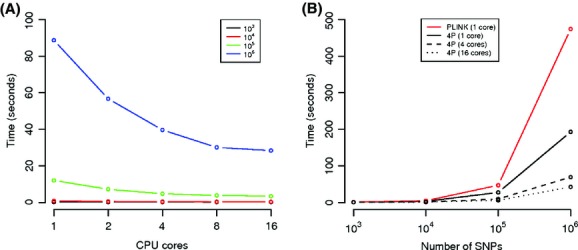
*4P* execution times. (A) The time required by *4P* to compute five different pairwise measures of genetic differentiation (see the main text for details) is reported as a function of the number of core; different lines correspond to datasets with different numbers of SNPs. (B) The time required by *4P* and *PLINK* to compute expected and observed heterozygosities is reported as a function of the data set size; *PLINK* is not implemented for multiple cores.

The time required by *4P* to compute all the between-population statistics and the comparison of this computing time and the number of cores and number of SNPs in the data set are shown in Fig.1A. For data sets up to 10,000 SNPs, the data processing (including file loading) required between 0.17 and 0.84 sec. Multiple cores did not improve the speed of the computation, due to parallel environment overhead. For larger data sets, the impact of parallel computing was evident in the analysis of 10^6^ SNPs, for example, the baseline serial time decreased from 88.70 sec to 56.69, 39.64, 30.08, and 28.38 with 2, 4, 8, and 16 cores, respectively. The increase of speed with increasing number of cores was less pronounced with 10^5^ SNPs, but it is important to note that even a small reduction of computing time is very important when summary statistics are computed from multiple simulated data sets.

Under our implementation, the decrease of computing time with the number of cores also rapidly reaches a plateau in the analysis of larger datasets. This result (considering that the maximum amount of RAM memory expended for the analysis of the largest dataset never exceeded 2GB) suggests that low- or medium-end computers are sufficient for most SNP-exploratory analyses.

The speed of *4P* was initially compared with that of *PLINK*, which allows the computation of observed and expected heterozygosity in large data sets of SNPs. When only a single core was used for the analysis, *4P* was 1.5 to 2.5 times faster than *PLINK*, depending on the number of SNPs analyzed (see Fig.1B). Using the multicore option in *4P* (not implemented in *PLINK*), the execution times decreased substantially. For example, with a data set of 10^6^ SNPs and 4 or 16 cores, *4P* accomplishes all the computations in 69.2 and 42.3 sec, respectively, about 7 and 11 times faster than *PLINK*, respectively. Again, a moderate improvement was detected with small datasets (10^3^ and 10^4^) due to the parallel environment overhead. To validate this conclusion, we additionally analyzed a real data set from the human chromosome 1 produced by the 1000 Genomes Project, Phase I (available at ftp://ftp.1000genomes.ebi.ac.uk/vol1/ftp/release/20110521/). This data set, stored in a *ped* file, include 1092 individuals and 3 × 10^6^ SNPs. *4P* (with 16 cores) is about three times faster than *PLINK* when observed and expected heterozygosites are computed. In particular, PLINK required 29 min and 45 sec to load and run the analysis, and *4P* only 9 min and 9 sec. The difference between *4P* and *PLINK* is reduced in this experiment compared to the analysis of simulated data sets, due to the much slower loading step of *ped* compared to *arp* files. When only the computation time is considered, the speed difference between *4P* and *PLINK* is similar to that observed in simulated data (about 11X, with 16 cores).

We then compared *4P* with *PopGenome* (Pfeifer et al. 2014), a set of R functions recently developed to handle whole-genome data. Our goal was to detect the differences between the two packages in reading a real data set of one million of SNPs (as above, located in the chromosome 1 and typed in 1092 human individuals for the 1000 Genomes Project) and in computing genetic variation indices. We used the *PopGenome readVCF* function that is optimized for loading compressed and tabix indexed *vcf* files. 4P was able to load the SNP data set in less than half of the time required by the best optimized *readVCF* function in *PopGenome* (5 min vs. 11 min; the time required by *PopGenome* was in agreement with that reported in Pfeifer et al. 2014). We then compared the performances of the two packages in computing some genetic diversity indices. Due to the very large memory requirement of *PopGenome*, it was not possible to analyze the whole SNP data set in a single step. We therefore proceeded subdividing initially the region of the human Chromosome 1 containing one million SNP in nonoverlapping Windows of 1,000 base pairs. *PopGenome* completed the computation of the nucleotide/haplotype diversity using the *diversity.stat* function in about 10 min (in agreement with the performances reported in Pfeifer et al. 2014), using 8GB of memory. *4P* computed the observed and expected heterozigosity, and the allele frequency spectrum in 2′30″, 47″, and 33″ using 1, 4, or 8 cores, respectively, and never consuming more than 3 GB of memory.

Finally, we compared *4P* with the *adegenet* R package using a single core, which is the only implementation of the functions in *adegenet* that compute genetic variation measures. Even with a single core, the C code in 4P was much faster than the R code in *adegenet*. Detailed results are not shown, but with the largest datasets, the computation time dropped from hours (*adegenet*) to minutes (*4P*).

## Conclusions

We introduce *4P*, an efficient C program for the parallel analysis of large SNP data sets. Several population genetics statistics within and between populations can be computed from real or simulated data set. *4P* is faster or much faster than comparable packages, do not require *ad hoc* scripts used sometimes to parallelize serial programs and collect the data (although this strategy can be used also with 4P to additionally increase its speed), and it can be used in stand-alone computers and servers.

## Data Accessibility

The manual, the source code, the program (for Unix, MacOs and Windows), and some example files are available at https://github.com/anbena/4p under the GPLv3 license.

## References

[b1] Abecasis GR, Auton A, Brooks LD, DePristo MA, Durbin RM, Handsaker RE (2012). An integrated map of genetic variation from 1,092 human genomes. Nature.

[b2] Barnett DW, Garrison EK, Quinlan AR, Stromberg MP, Marth GT (2011). BamTools: a C++ API and toolkit for analyzing and managing BAM files. Bioinformatics.

[b3] Catchen J, Hohenlohe PA, Bassham S, Amores A, Cresko WA (2013). Stacks: an analysis tool set for population genomics. Mol. Ecol.

[b4] Danecek P, Auton A, Abecasis G, Albers CA, Banks E, DePristo MA (2011). The variant call format and VCFtools. Bioinformatics.

[b5] Davey JW, Hohenlohe PA, Etter PD, Boone JQ, Catchen JM, Blaxter ML (2011). Genome-wide genetic marker discovery and genotyping using next-generation sequencing. Nat. Rev. Genet.

[b6] Excoffier L, Foll M (2011). fastsimcoal: a continuous-time coalescent simulator of genomic diversity under arbitrarily complex evolutionary scenarios. Bioinformatics.

[b7] Excoffier L, Dupanloup I, Huerta-Sánchez E, Sousa VC, Foll M (2013). Robust Demographic Inference from Genomic and SNP Data. PLoS Genet.

[b8] Gutenkunst RN, Hernandez RD, Williamson SH, Bustamante CD (2009). Inferring the Joint Demographic History of Multiple Populations from Multidimensional SNP Frequency Data. PLoS Genet.

[b9] Hedrick PW (2005). A standardized genetic differentiation measure. Evolution.

[b10] Hess JE, Campbell NR, Close DA, Docker MF, Narum SR (2013). Population genomics of Pacific lamprey: adaptive variation in a highly dispersive species. Mol. Ecol.

[b11] Hohenlohe PA, Day MD, Amish SJ, Miller MR, Kamps-Hughes N, Boyer MC (2013). Genomic patterns of introgression in rainbow and westslope cutthroat trout illuminated by overlapping paired-end RAD sequencing. Mol. Ecol.

[b12] Jombart T, Ahmed I (2011). adegenet 1.3-1: new tools for the analysis of genome-wide SNP data. Bioinformatics.

[b13] Jost L (2008). GST and its relatives do not measure differentiation. Mol. Ecol.

[b14] Keller I, Wagner CE, Greuter L, Mwaiko S, Selz OM, Sivasundar A (2013). Population genomic signatures of divergent adaptation, gene flow and hybrid speciation in the rapid radiation of Lake Victoria cichlid fishes. Mol. Ecol.

[b15] Li H, Handsaker B, Wysoker A, Fennell T, Ruan J, Homer N (2009). The Sequence Alignment/Map format and SAMtools. Bioinformatics.

[b16] Nei M (1973). Analysis of gene diversity in subdivided populations. Proc. Natl Acad. Sci. USA.

[b17] Nei M, Chesser RK (1983). Estimation of fixation indices and gene diversities. Ann. Hum. Genet.

[b18] Ogden R, Gharbi K, Mugue N, Martinsohn J, Senn H, Davey JW (2013). Sturgeon conservation genomics: SNP discovery and validation using RAD sequencing. Mol. Ecol.

[b19] Pfeifer B, Wittelsbuerger U, Ramos-Onsins SE, Lercher MJ (2014). PopGenome: an efficient Swiss army knife for population genomic analyses in R. Mol. Biol. Evol.

[b20] Purcell S, Neale B, Todd-Brown K, Thomas L, Ferreira MA, Bender D (2007). PLINK: a tool set for whole-genome association and population-based linkage analyses. Am. J. Hum. Genet.

[b21] The Heliconius Genome Consortium (2012). Butterfly genome reveals promiscuous exchange of mimicry adaptations among species. Nature.

[b22] Wagner CE, Keller I, Wittwer S, Selz OM, Mwaiko S, Greuter L (2013). Genome-wide RAD sequence data provide unprecedented resolution of species boundaries and relationships in the Lake Victoria cichlid adaptive radiation. Mol. Ecol.

[b23] Weir BS, Cockerham CC (1984). Estimating F-Statistics for the Analysis of Population Structure. Evolution.

